# The first wave of COVID-19 in Malta; a national cross-sectional study

**DOI:** 10.1371/journal.pone.0239389

**Published:** 2020-10-15

**Authors:** Sarah Micallef, Tonio V. Piscopo, Ramon Casha, Denise Borg, Chantal Vella, Maria-Alessandra Zammit, Janice Borg, Daniela Mallia, James Farrugia, Sarah Marie Vella, Thelma Xerri, Anette Portelli, Manuel Fenech, Claudia Fsadni, Charles Mallia Azzopardi

**Affiliations:** 1 Department of Medicine, Mater Dei Hospital, Msida, Malta; 2 Department of Infectious Diseases, Mater Dei Hospital, Msida, Malta; 3 Department of Pharmacy, Mater Dei Hospital, Msida, Malta; Laboratoire National de Santé, LUXEMBOURG

## Abstract

**Introduction:**

The COVID-19 pandemic has posed major challenges to all aspects of healthcare. Malta’s population density, large proportion of elderly and high prevalence of diabetes and obesity put the country at risk of uncontrolled viral transmission and high mortality. Despite this, Malta achieved low mortality rates compared to figures overseas. The aim of this paper is to identify key factors that contributed to these favorable outcomes.

**Methods:**

This is a retrospective, observational, nationwide study which evaluates outcomes of patients during the first wave of the pandemic in Malta, from the 7^th^ of March to the 24^th^ of April 2020. Data was collected on demographics and mode of transmission. Hospitalization rates to Malta’s main general hospital, Mater Dei Hospital, length of in-hospital stay, intensive care unit admissions and 30-day mortality were also analyzed.

**Results:**

There were 447 confirmed cases in total; 19.5% imported, 74.2% related to community transmission and 6.3% nosocomially transmitted. Ninety-three patients (20.8%) were hospitalized, of which 4 were children. Patients with moderate-severe disease received hydroxychloroquine and azithromycin, in line with evidence available at the time. A total of 4 deaths were recorded, resulting in an all-cause mortality of 0.89%. Importantly, all admitted patients with moderate-severe disease survived to 30-day follow up.

**Conclusion:**

Effective public health interventions, widespread testing, remote surveillance of patients in the community and a low threshold for admission are likely to have contributed to these favorable outcomes. Hospital infection control measures were key in preventing significant nosocomial spread. These concepts can potentially be applied to stem future outbreaks of viral diseases. Patients with moderate-severe disease had excellent outcomes with no deaths reported at 30-day follow up.

## Introduction

COVID-19 is caused by a novel coronavirus SARS-CoV-2, a single-stranded RNA-enveloped virus, which emerged from Wuhan, China in late December 2019. By end of February 2020, many European countries had reported viral transmission. By the 24th of April 2020, 209 countries had reported cases of COVID-19, with the United States reporting the highest number of cases, followed by Spain, Italy, Germany, the United Kingdom and France. More than 2.84 million cases had been diagnosed globally by this date, with over 200,000 fatalities [1].

Malta is one of the smallest nations in Europe with a population of 493,559 [2] and a population density of 1562 persons/km2. The high population density and proximity to Italy, one of the hardest hit countries worldwide, put Malta at high risk of SARS-CoV-2 transmission. Furthermore, the high prevalence of hypertension (22% in males, 23% in females) [3], obesity (36.9% males, 31.25% females) [4], type 2 diabetes mellitus (10.3%) [5] along with a significant elderly population (18.7%) [6] put Malta at a particularly high risk of morbidity and mortality [7]. Furthermore, Malta has only one main general hospital, Mater Dei Hospital (MDH), which meant that healthcare services could very easily be overwhelmed.

The first case of COVID-19 was reported in Malta on the 7^th^ of March 2020. Despite the risk factors, Malta was successful at achieving low mortality rates compared with figures overseas. By June 2020, Malta registered a death rate of 19 deaths per million population, comparing very favorably with international figures [8]. The situation in Malta remains stable as of July 2020, with no further COVID-19 related deaths reported, although there has been a recent spike in imported cases in view of border reopening and mass gatherings.

A number of factors, including public health interventions, infection control measures at MDH and management pathways of COVID-19 patients are likely to have contributed to these figures [9]. This is an observational, nationwide study looking at patient demographics, hospitalization rates and mortality outcomes during the first wave of COVID-19 in our country. This study aims to identify key factors that are likely to have contributed towards Malta’s successful outcomes.

## Methods

### Data collection

Patients diagnosed with COVID-19 from the 7^th^ of March to the 24^th^ of April 2020, during the first wave of the pandemic in Malta, were analyzed for demographic data including age and gender as well as mode of transmission. Data was collected using online resources published by the Superintendence of Public Health. Hospitalization rates and 30-day mortality were also collected.

An in-depth retrospective sub-analysis on adult patients requiring admission to MDH during the same time period was also performed. Data was collected on patient demographics, as well as comorbidities, clinical severity of disease, laboratory parameters on admission, admission to an intensive care unit (ICU), treatment administered and all-cause mortality. Patients were followed up for 30 days after medical discharge. Data was collected by reviewing patient files and electronic records, clinical and laboratory parameters and corrected QT (QTc) measurement on ECG.

### Inclusion and exclusion criteria

Only patients with a diagnosis confirmed by polymerase-chain reaction (PCR) using the Applied Biosystems™ 7500 Real-Time PCR platform and MWE Medical Wire® nasopharyngeal swabs were included in the study. Patients with a suspected or probable diagnosis of COVID-19 as defined by international criteria [10] with a negative PCR swab were excluded from this study. Patients under the age of 16 years of age were excluded from the sub-analysis on our inpatient cohort.

### Sub-analysis on inpatient cohort

**Fig 1:**
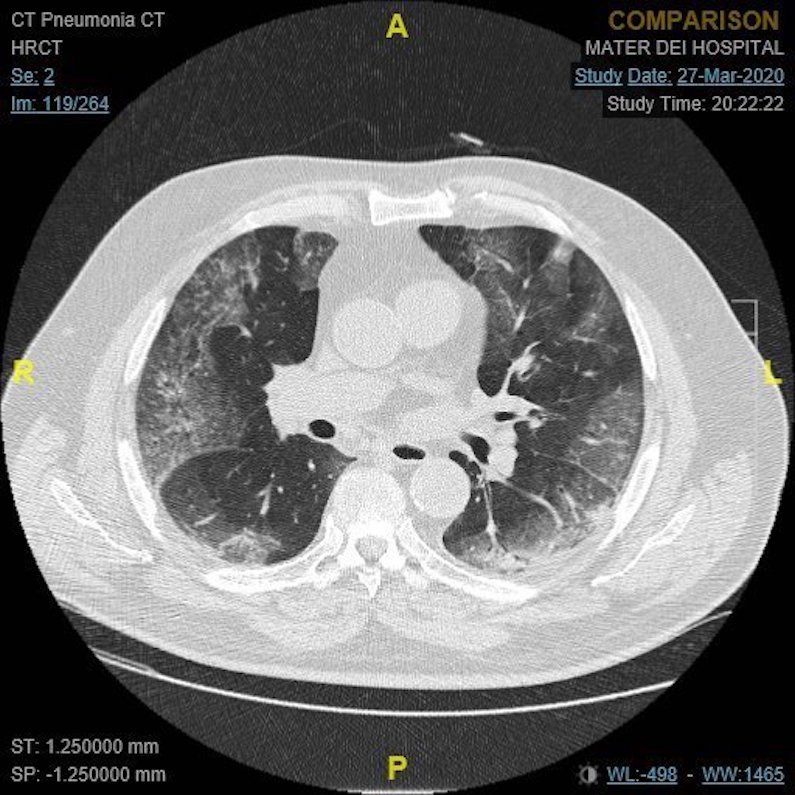
A non-contrast CT showing extensive ground-glass changes in both lung lobes with predominant peripheral distribution, compatible with moderate-severe bilateral COVID-19 pneumonia.

Oxygen saturations and radiological findings were considered as severity markers for patients requiring admission to hospital. Low oxygen saturations of ≤ 93% on room air and/or significant radiological findings were considered to be markers of moderate-severe disease. Significant radiological findings included bilateral infiltrates on chest x-ray (CXR), or typical COVID-19 changes on non-contrast computed tomography (CT) of the chest, particularly ground-glass changes, crazy-paving appearance and airspace consolidations (Fig 1).

We compared age, gender distribution, comorbidity burden and laboratory parameters on admission in patients with mild disease and those with moderate-severe disease. Outcomes measured for our hospitalized adult cohort included all-cause 30-day mortality from medical discharge, admission to ICU, length of in-hospital stay, length of stay on ICU and adverse events on treatment.

### Data protection and ethical approval

This observational study was approved by the data protection officer and the chief executive officer of Mater Dei Hospital. After consultation with the Faculty of Medicine and Surgery Research Ethics Committee of the University of Malta, ethics approval was waived in view of the retrospective nature of the study.

### Statistical analysis

Statistical analysis was performed using Microsoft® Excel Analysis ToolPak and XLSTAT®. Tests for normality were performed using the Kolmogorov–Smirnov test. Numerical data was expressed in terms of means and standard deviations, or medians and interquartile ranges. Categorical data was expressed in terms of frequency and percentages. For continuous variables, unpaired T-test was used for parametric data, while the Mann-Whitney U test was used for non-parametric data. Chi-square test and Fisher’s exact test were used to compare categorical variables. A p-value of 0.05 or less was considered significant.

## Results

**Fig 2:**
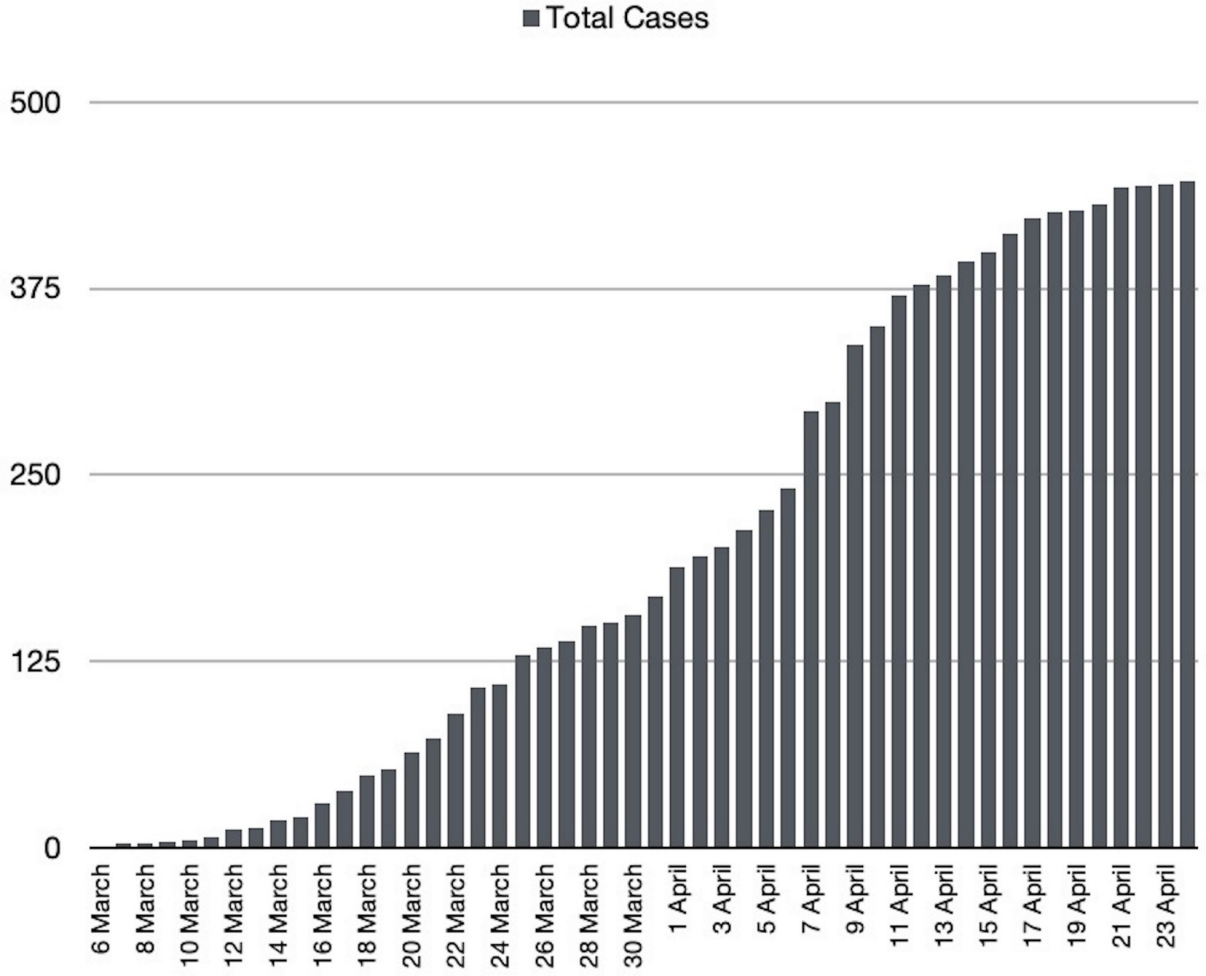
Number of confirmed cases of COVID-19 in Malta [10].

By the 24^th^ of April, at least 27,422 people had been tested for COVID-19 in Malta, corresponding to a rate of around 55,560 tests per million population [11], the third highest rate of testing worldwide. There were 447 confirmed cases reported across the Maltese Islands (Fig 2) corresponding to a rate of 1012 total cases per million population. These included 223 patients who had recovered [12].

The first case, reported on 7^th^ March, was related to travel and subsequently another 86 imported cases (19.5%) were identified. Community transmission, first noted on the 16^th^ of March 2020, accounted for the majority of cases (332 cases, 74.2%), while 28 cases (6.3%) were secondary to nosocomial transmission. The highest proportion of cases (16.3%) occurred amongst individuals aged 30–34 years. Only 8.5% of cases were under 20 years of age. Males constituted 59.3% of cases.

### Inpatient cohort

Out of 447 patients with confirmed COVID-19 diagnosis in Malta, 93 patients were admitted to MDH, corresponding to a hospitalization rate of 20.8%. Four patients were under 16 years of age and were excluded from the study. The average age in our adult admitted cohort (n = 89) was 50.3 years (S.D. 20.2 years). Sixty patients (67.4%) were males. Nineteen patients (21.3%) suffered from hypertension, 11 (12.4%) from diabetes, 13 (14.6%) from cardiovascular disease and 13 (14.6%) from chronic lung conditions. Eighteen patients (20.2%) had two or more comorbidities. No patients were lost to follow-up.

**Fig 3:**
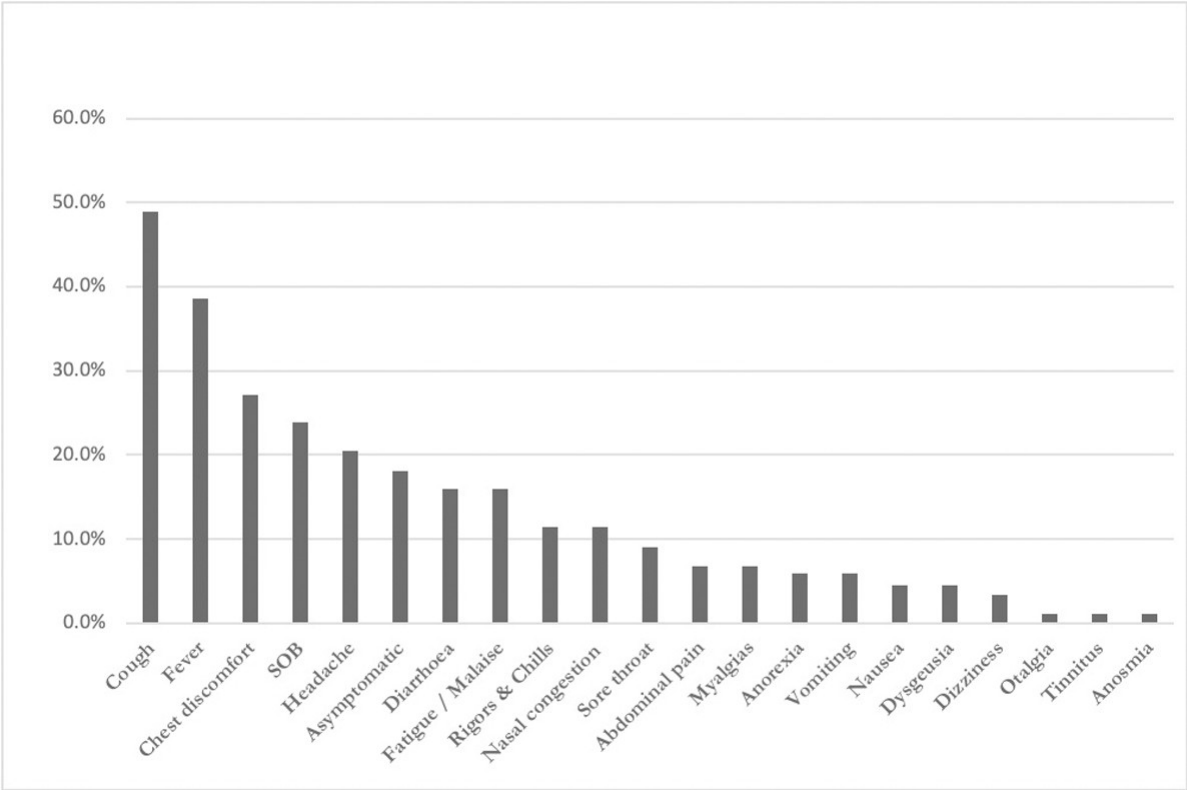
Symptomatology of COVID-19 infection in admitted patients (n = 89).

Forty-three patients presented with a cough (48.3%), 35 with fever (39.3%), 24 with chest discomfort (27.0%) and 21 with shortness of breath (23.6%). Sixteen patients (18.0%) were asymptomatic at the time of diagnosis and were diagnosed during screening for elective procedures (Fig 3). Five of these (5.62%) were pregnant females in their third trimester who were identified on admission for elective caesarean section. All patients delivered without any complications.

**Fig 4:**
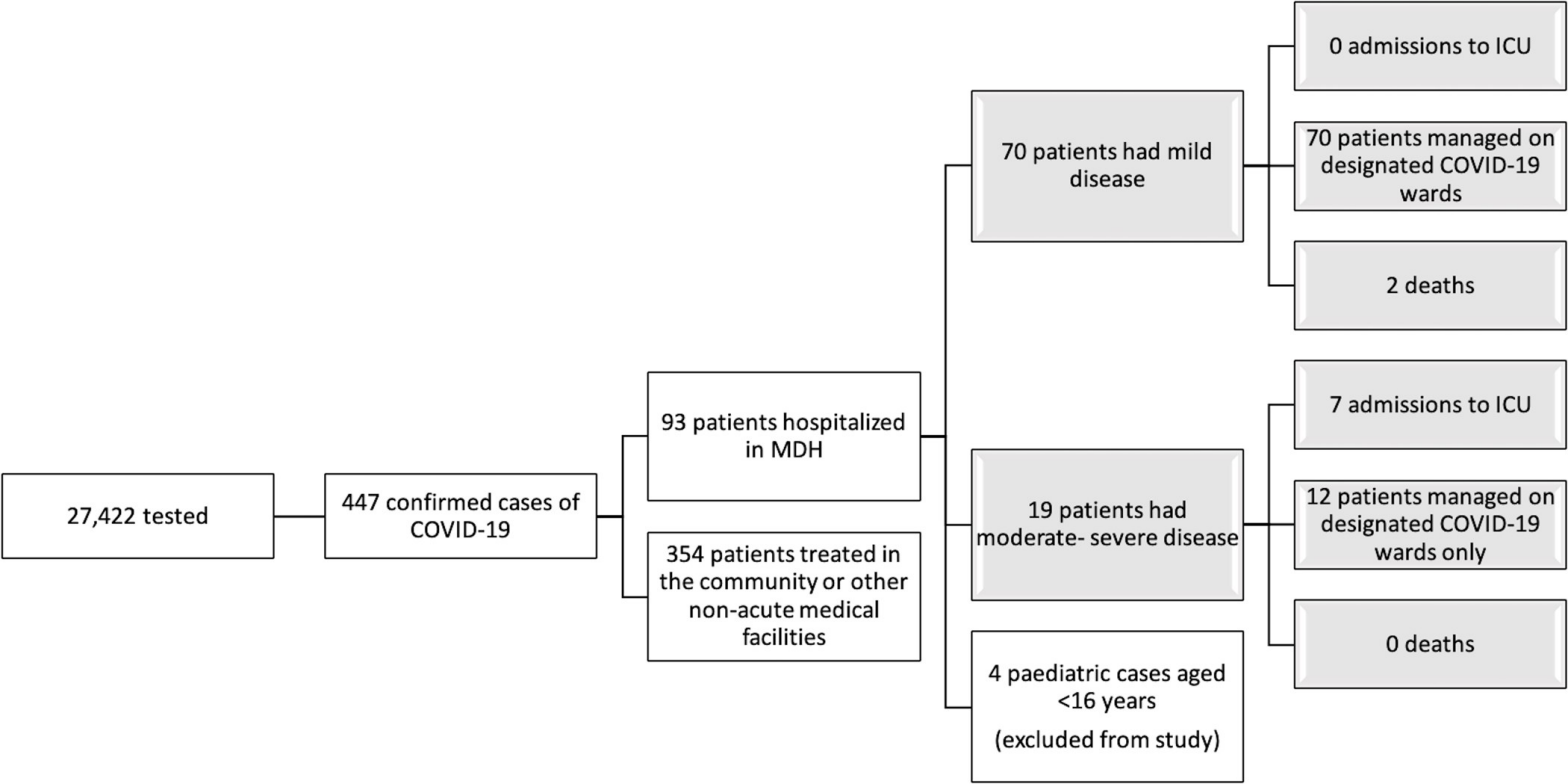
Hospitalized patients during the first wave COVID-19 in Malta; study cohort highlighted in grey. Abbreviations: MDH, Mater Dei Hospital; ICU, Intensive Care Unit.

Nineteen patients (21.3%) had moderate-severe disease while seventy patients (78.7%) had only mild disease (Fig 4). Those with moderate-severe disease were significantly older than those with mild disease (61.5±15.7 years vs 47.2±20.3 years, p = 0.005). There was no significant difference in gender distribution between the two cohorts (p = 0.227). Furthermore, patients with moderate-severe disease tended to have more comorbidities compared to those with mild disease, however this did not reach statistical significance (Table 1).

**Table 1:** Characteristics of our admitted cohort, comparing those with moderate-severe disease and those with mild disease.

	No. (%)			
Characteristics	Total (n = 89)	Patients with moderate-severe disease (n = 19)	Patients with mild disease (n = 70)	P-value
**Demographics**				
Age, mean, [SD], years	50.3 [20.2]	61.5 [15.7]	47.2 [20.3]	0.005
Male	60 (67.4)	15 (78.9)	45 (64.3)	0.23
**Comorbidities**				
Hypertension	19 (21.3)	6 (31.6)	13 (18.6)	0.22
Diabetes Mellitus	11 (12.4)	4 (21.1)	7 (10.0)	0.19
Cardiovascular Disease	13 (14.6)	5 (26.6)	8 (11.4)	0.10
Chronic Lung Disease	13 (14.6)	3 (15.5)	10 (14.3)	0.87
≥ 2 Comorbidities	18 (20.2)	6 (31.6)	12 (17.1)	0.17
**Laboratory Parameters on Admission**			*	
Lymphocyte count, median, [IQR], 10^9/L	1.38 [0.35–1.78]	1.02 [0.80–1.46]	1.44 [1.11–1.93]	0.002
Eosinophil count, median, [IQR], 10^9/L	0.05 [0.00–0.11]	0.02 [0.00–0.06]	0.06 [0.02–0.14]	0.001
C-reactive protein, median, [IQR], mg/L	16.0 [3.3–50.5]	52.1 [32.4–73.9]	7.6 [2.0–30.2]	<0.001
**Patient Outcomes**				
Admission to ICU	7 (7.9)	7 (36.8)	0 (0.0)	<0.001
Length of admission in ICU, median, [IQR], days	4.0 [3.0–7.0]	4.0 [3.0–7.0]	---	---
Length of in-hospital stay, median [IQR], days	3.0 [2.0–6.0]	9.0 [5.5–14.5]	3.0 [2.0–5.0]	<0.001
30-day mortality	2 (2.2)	0 (0.0)	2 (2.9)	1.00

Abbreviations: SD, Standard Deviation; IQR, Interquartile Range; ICU, Intensive Care Unit.

*Laboratory parameters on admission were only available for 51 out of 70 patients with mild diseases.

Laboratory parameters were available for all 19 patients with moderate-severe disease, and for 51 patients with mild disease (Table 1). Lymphocyte count on admission was significantly lower in patients with moderate-severe disease compared to those with mild disease with similar differences noted in eosinophil count on admission. C-Reactive protein (CRP) on admission was significantly higher in those with moderate-severe disease.

Patients with mild disease were managed with supportive therapy alone. All patients with moderate-severe disease were managed with supportive therapy as well as combination therapy, based on the literature available at the time [13]. This consisted of a combination of hydroxychloroquine (HCQ) and azithromycin (AZI) with close ECG monitoring.

No significant adverse events were observed in the HCQ+AZI cohort. All patients had a QTc <460ms prior to starting treatment, with no statistically significant difference in QTc on repeat ECG testing (p = 0.82). No patients suffered from arrhythmias or significant QTc prolongation (to more than >500ms or increase in QTc by >60ms from baseline) after starting treatment.

The median length of in-hospital stay was 9.0 days (IQR 5.5–14.5) for patients with moderate-severe disease, and 3.00 days (IQR 2.0–5.0) for those with mild disease (p = <0.001). None of the patients with mild disease needed ICU admission. Conversely, seven patients (36.8%) out of those with moderate-severe disease required ICU admission; one patient required invasive ventilation. The median length of admission on ICU was 4.0 days (IQR 3.0–7.0).

During the follow-up period, a total of four deaths were recorded (n = 447) giving rise to an all-cause mortality rate of 0.89%. Two of these deaths were recorded at MDH; both were deemed to have mild COVID-19 disease. One death occurred in an 84-year-old male with multiple comorbidities. The second death occurred in a 96-year-old female who died of an unrelated surgical cause, despite testing positive for COVID-19. The other two deaths occurred in elderly patients, one in a subsidiary hospital on the sister island of Gozo and the other in a rehabilitation hospital. Both had multiple comorbidities and were only managed conservatively. Importantly, all patients who were admitted to MDH with moderate-severe disease (including those who required ICU admission) were successfully discharged from hospital, with no deaths recorded at 30-day follow-up.

## Discussion

The COVID-19 pandemic is a rapidly developing global emergency which has posed major challenges to all aspects of healthcare. Quick adaptation and pragmatic measures are vital in dealing with such challenges. Extensive research is crucial to expand our understanding of the disease, improve our ability to reduce transmission and identify those at higher risk of deterioration. There is a great need for effective therapeutics backed by robust clinical evidence.

Malta’s population density, together with a high prevalence of diabetes, obesity and hypertension and a significant proportion of elderly individuals put the country at particular risk of uncontrolled viral transmission and high mortality. Furthermore, having only one major acute hospital in the country meant that healthcare facilities could easily be overwhelmed.

Despite all these factors, Malta achieved favorable overall mortality rates compared to figures overseas [14]. At the time of writing, Malta has recorded a total of 19 deaths per million population [8]. From a public health perspective, various measures are likely to have contributed to such low numbers, and may therefore be applied to future outbreaks of viral diseases.

Firstly, a number of effective public health interventions were implemented. Schools and childcare centers were closed within a week of the first infected case, with this likely to have had a significant impact in reducing exposure and spread amongst children and adolescents. In fact, while persons under 20 years of age constitute 18.6% of the Maltese population [6], only 8.5% of cases were reported to occur in this cohort. Furthermore, a partial lockdown for vulnerable groups (immunosuppressed persons, those with chronic medical conditions particularly moderate-severe lung dysfunction, pregnant females, and persons above 65 years of age) was recommended, with people encouraged to work from home [15].

A two-week quarantine for all returning travelers was imposed, and borders were closed within two weeks of the first reported case. All confirmed cases were isolated, and a robust contact-tracing system allowed the identification of contacts who were placed under a mandatory two-week quarantine. Quarantine was enforced by the country’s armed forces, with fines issued to individuals caught breaking quarantine. Individuals were allowed to self-quarantine in their homes, with designated centers being available for those who were unable to do so for logistical reasons. Social distancing was also enforced with fines.

Use of facemasks was not made obligatory by the 24^th^ of April but became mandatory in shops and aboard public transport once restrictions started to be eased in May 2020.

Secondly, testing for COVID-19 was also made widely accessible, with testing hubs strategically placed all over the islands. Apart from testing symptomatic individuals, screening was also available for asymptomatic persons and healthcare workers. Furthermore, all urgent and elective admissions to MDH, were screened for COVID-19 irrespective of their presenting complaint, and isolated or quarantined accordingly. These measures prevented significant nosocomial spread amongst patients and healthcare workers. In the community, the detection of asymptomatic infected persons allowed timely implementation of 14-day-quarantine of close contacts preventing further viral transmission.

By the 24^th^ of April 2020, 55,560 tests per million population had been carried out, one of the highest rates of testing worldwide. Over a thousand tests were performed every day, indicating a good uptake of testing by the Maltese population. This persists until the time of writing (1^st^ August), with 291,518 tests per million population having been performed since the beginning of the pandemic. With such a high rate of testing, it is likely that the reported number of cases reflect the true disease burden in the community, with few cases of COVID-19 being missed.

The timeliness and rigorousness of these public health measures is likely to have been crucial in stemming the pandemic. This has also been observed in other countries with robust public health systems [16]. On the other hand, lack of vigorous case identification and contact-tracing leads to unchecked viral transmission and consequent overloading of healthcare systems and fatalities [17].

Infection control measures applied to the main general hospital, MDH, were also crucial in preventing significant nosocomial spread. These measures included the routine screening of all elective and emergency admissions to hospital for COVID-19, provision of adequate personal protective equipment to all healthcare staff and the restructuring and repurposing of various wards at MDH. All emergency admissions were held in dedicated transition areas pending result of their COVID-19 swab prior to transfer to general medical or surgical wards. Patients with confirmed COVID-19 infection were managed in an infectious disease unit and a dedicated intensive care unit. Medical practice was shifted to a ward-based, rather than a firm-based system, with the intention to contain any potential outbreaks on the wards. Furthermore, in-hospital visits were suspended to prevent nosocomial spread. Mandatory temperature screening was enforced for all healthcare professionals and outpatients.

Another reason to explain the low case fatality rate is the management of patients once diagnosed with COVID-19. This included the provision of rigorous, remote monitoring of patients diagnosed with COVID-19 in the community. These patients were assessed on diagnosis and daily thereafter by a community team of doctors using a standardized protocol. This would identify patients at higher risk of deterioration, prompting early referral to hospital if significant symptoms or comorbidities were present. This also explains why there was no significant difference observed in the prevalence of comorbidities when comparing admitted patients with mild disease and those with moderate-severe disease. The low threshold for hospital admission meant that a large proportion of patients were admitted to hospital due to their comorbidities irrespective of their COVID-19 disease severity.

The management protocol used for admitted patients with COVID-19 is also likely to have played a significant role in patient outcomes, particularly for patients with moderate-severe disease. Once admitted to hospital, patients were stratified according to disease severity, using hypoxia and radiological findings as markers of severity. This stratification was based on published international guidelines used at the time [18, 19]. Patients with moderate-severe disease were flagged to the COVID-19 ICU on admission, facilitating transfer if and when patients deteriorated. Our study also shows that CRP, lymphocyte and eosinophil counts on admission reflect disease severity and can therefore be useful indices of severity.

At our center, patients with moderate-severe disease were treated with a combination of HCQ (400mg twice daily on day 1 followed by 200mg twice daily on days 2–5) and AZI (500mg daily on day 1 followed by 250mg daily on days 2–5). These local guidelines mirrored a number of international guidelines [19–21], and were in line with the literature available at the time [13, 22]. The initial claims of the degree of effectiveness of HCQ+AZI [13] were later refuted by a study which found no strong antiviral activity or clinical benefit of combination therapy of HCQ+AZI [23]. Similar outcomes were observed in a pilot study conducted in Shanghai, using HCQ as monotherapy [24]. An observational study carried out on 1376 patients in the United States showed that hydroxychloroquine was not associated with any benefit on intubation or death, however highlighted the need for randomized control studies [25]. More recent literature showed that hydroxychloroquine was associated with decreased in-hospital survival and an increased frequency of arrhythmias when used for COVID-19 [26]. This led to temporary suspension of the HCQ arm within the Solidarity trial, however this was resumed again on June 3^rd^ 2020 after reassessment of the data [27].

Patients with moderate-severe disease had excellent outcomes with no deaths recorded and no adverse events noted on treatment. It is not possible to draw any conclusions from these results, due to absence of a control-arm and the small number of patients. Possible reasons for the positive outcomes include the widespread availability of testing allowing early confirmation of diagnosis after onset of symptoms, and the low threshold for admission meaning that those with moderate-severe disease were identified without delay and management instituted in a timely manner. Furthermore, close ECG monitoring for QTc prolongation may have mitigated the increased risk of arrhythmia with combination therapy.

To our knowledge, this is the only study that describes a country’s holistic approach to the COVID-19 pandemic, including public health measures, hospital infection control policies and patient care pathways. The limitations of this study are its retrospective and observational nature.

## Conclusion

This nationwide study aims to identify factors that contributed towards Malta’s favorable outcomes during the first wave of the COVID-19 pandemic. Timely and effective public health interventions and widespread access to testing for COVID-19 undoubtedly played an important role in curbing the spread of COVID-19. Thorough infection control policies at MDH are likely to have significantly prevented nosocomial spread and decimation of the workforce.

A rigorous and remote surveillance system for patients diagnosed in the community and a low threshold for admission meant that patients with moderate-severe disease were identified early and managed accordingly. Patients admitted to hospital with moderate-severe disease had successful outcomes, with all patients discharged home and no deaths reported in this group.
