# Correction: The first wave of COVID-19 in Malta; a national cross-sectional study

**DOI:** 10.1371/journal.pone.0255881

**Published:** 2021-08-03

**Authors:** Sarah Micallef, Tonio V. Piscopo, Ramon Casha, Denise Borg, Chantal Vella, Maria-Alessandra Zammit, Janice Borg, Daniela Mallia, James Farrugia, Sarah Marie Vella, Thelma Xerri, Anette Portelli, Manuel Fenech, Claudia Fsadni, Charles Mallia Azzopardi

There is an error in the thirteenth paragraph of the Discussion section of this article [1]. Prior to the publication of this article [1], reference 26 was retracted by The Lancet and should not have been cited. The following text in the thirteenth paragraph of the Discussion should be removed: “More recent literature showed that hydroxychloroquine was associated with decreased in-hospital survival and an increased frequency of arrhythmias when used for COVID-19 [26]. This led to temporary suspension of the HCQ arm within the Solidarity trial, however this was resumed again on June 3rd 2020 after reassessment of the data [27].” As a result of this correction to the Discussion text, references 26–27 are no longer cited in the article. The authors are not aware of further studies that associate hydroxychloroquine with decreased in-hospital survival.

The Data Availability statement for this paper is incorrect. The correct statement is: COVID-19 case data were sourced from the publicly available Superintendence of Public Health, Malta (https://infogram.com/covid-19-malta-superintendence-of-public-health-1h7k23gorvzv4xr) and the Malta Health Promotion & Disease Prevention Directorate (https://geosys-mt.maps.arcgis.com/apps/opsdashboard/index.html#/c17848aba0f94d79a4f57a7a584c4e8e) following the protocol outlined in the Methods section. A de-identified dataset underlying the clinical results is available from the Dryad repository (doi: 10.5061/dryad.mcvdnck12).
